# A case of postoperative hepatic granuloma presumptively caused by surgical staples/clipping materials

**DOI:** 10.1186/s13000-015-0291-3

**Published:** 2015-07-09

**Authors:** Yasuhiro Nihon-Yanagi, Takao Ishiwatari, Yuichiro Otsuka, Yoichiro Okubo, Naobumi Tochigi, Megumi Wakayama, Tetsuo Nemoto, Manabu Watanabe, Hironori Kaneko, Yasukiyo Sumino, Kazutoshi Shibuya

**Affiliations:** Department of Surgical Pathology, Toho University School of Medicine, 6-11-1 Omori-Nishi, 143-8541 Ota-Ku, Tokyo Japan; Department of General and Gastroenterological Surgery, Toho University School of Medicine, 6-11-1 Omori-Nishi,, 143-8541 Ota-Ku, Tokyo Japan; Division of Gastroenterology and Hepatology; Department of Internal Medicine, Toho University School of Medicine, 6-11-1 Omori-Nishi, 143-8541 Ota-Ku, Tokyo Japan

**Keywords:** Hepatic granuloma, Metal hypersensitivity, Staple, Stapler, Titanium

## Abstract

A 66-year-old man with postsigmoidectomy status for colon cancer received laparoscopic partial hepatectomy due to a hepatic mass with employing titanium clips were for a vascular clamp. Histological examination showed liver metastasis from sigmoid colon cancer. Twenty-nine months after the partial hepatectomy, a mass developed on the stump at the hepatic resection. Laparoscopic left lateral segmentectomy was conducted under suspicion of cancer recurrence and an automatic titanium stapling device was used. The macroscopically cut surface of the liver showed a grey-white solid nodule measuring 23 x 20 mm and involving metal clips. The nodule was consistent with granuloma microscopically. Twenty-three months after the segmentectomy, a mass reappeared on the hepatic radial margin and an open left lateral hepatic lobectomy was performed because of its growth tendency. Histopathological examination revealed granuloma similar to the previous instance. Since these nodules formed a granulomatous lesion surrounding metal staples/clips and evidence of caseous necrosis was lacking, granuloma due to surgical staples/clips was suspected. Sporadic case reports of postoperative pulmonary granuloma at the staple line have been published previously, but there are no articles detailing a case involving hepatic granuloma. We present our case as the first report of postoperative staple-line hepatic granuloma.

## Background

It is important for clinicians to distinguish liver tumors detected after surgical intervention of malignant disorders from recurrent or metastatic lesions originating from the primary cancer.

Titanium is now widely used as a material in automatic stapling surgery because of its minimal irritability in the human body [1], but there are a number of case reports detailing significant allergic reactions caused by titanium [2–11]. Herein, we report a case of postoperative hepatic granuloma suspected of being caused by surgical staples/clipping materials.

## Case presentation

A 66-year-old man underwent a colectomy for sigmoid colon cancer who had been initiated on hemodialysis for end-stage renal failure of unknown cause since 54 years of age and had no history of allergic diseases. Pathological examination of the cancer ultimately demonstrated a stage II type according to TNM classification. He had no sequential adjuvant chemotherapy. Fifty-eight months after the colectomy, a nodule was detected at the left hepatic lobe. A partial hepatectomy was then performed. Histological examination showed liver metastasis of the sigmoid colon cancer. Surgeons used metal clips to control bleeding at the cut surface of the liver.

**Fig. 1:**
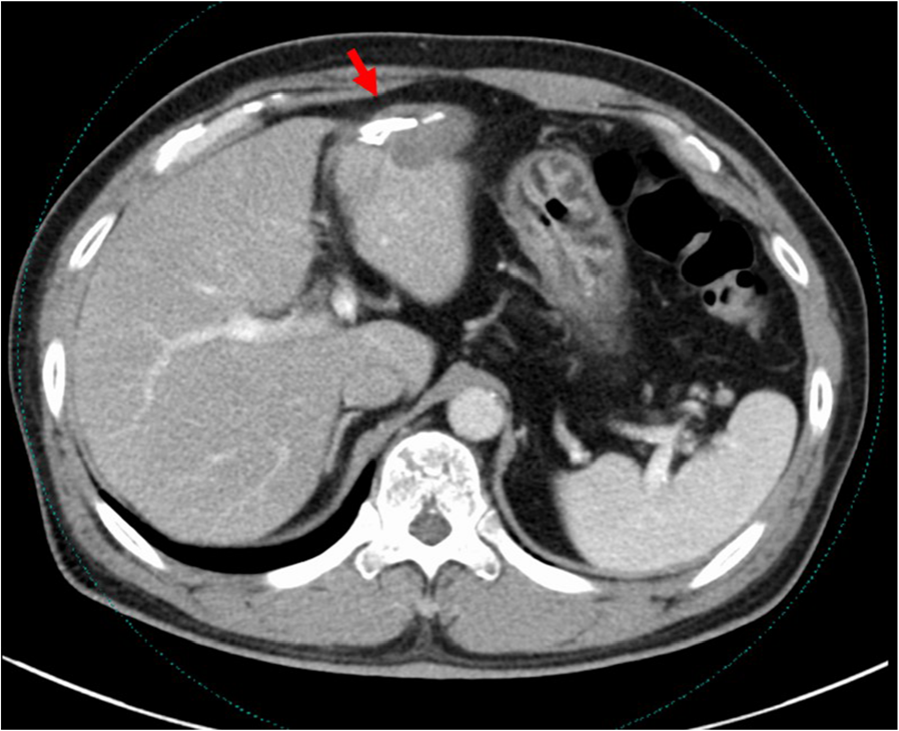
Contrast-enhanced CT scan. A non-enhanced mass (arrow) measuring 35 mm in length at the wedge from the cut stump of the hepatic resection

Twenty-nine months after the partial hepatectomy, complementary studies showed a mass lesion at the wedge from the cut stump at the hepatic resection. Computed tomography (CT) demonstrated a non-enhanced nodule measuring 35 mm in diameter (Fig. 1). Gray-scale ultrasound examination revealed a mass of 45-mm diameter containing a hyperechoic component. The mass remained a defect using contrast-enhanced ultrasonography with Sonazoid® throughout every phase. 18 F-fluorodeoxyglucose positron emission tomography (FDG-PET) and magnetic resonance imaging (MRI) were not utilized. Laparoscopic left lateral segmentectomy of the liver was performed with employing titanium alloy device (Echelon 60 Gold ETS Flex® 45, Ethicon Endo-Surgery, Inc. USA).

**Fig. 2:**
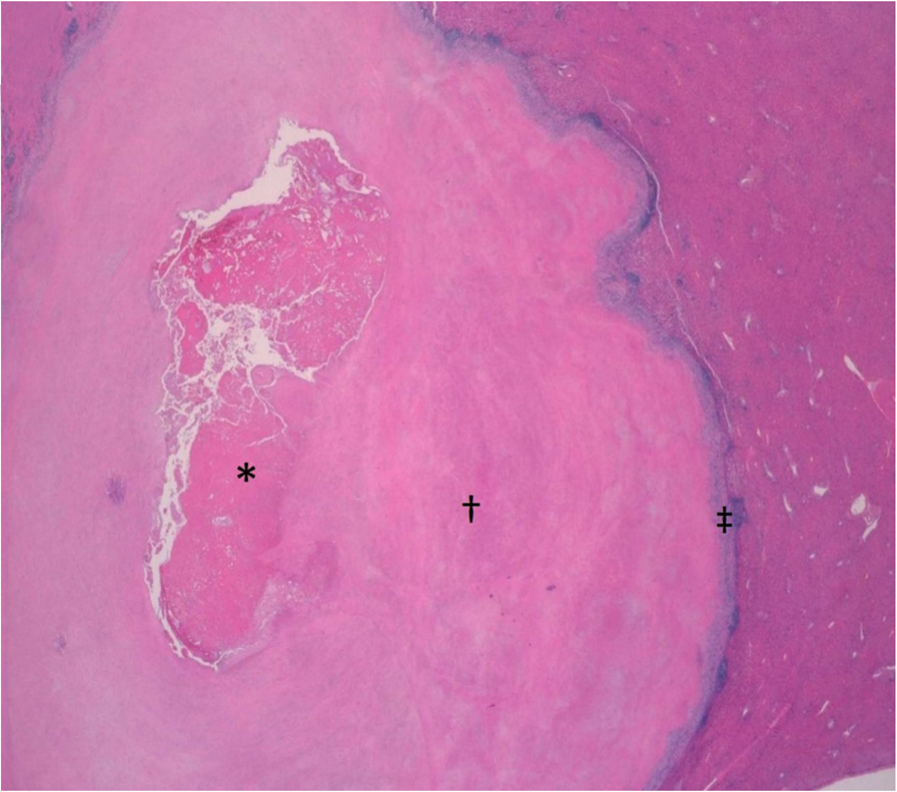
Histopathologic features. An overview loupe (hematoxylin-eosin: HE). The nodule had necrotic tissue in the center (*) surrounded by fibrous tissue **(†)** and circular formation of granuloma on the boundary between the hepatocytes and fibrous tissue **(‡)**

**Fig. 3:**
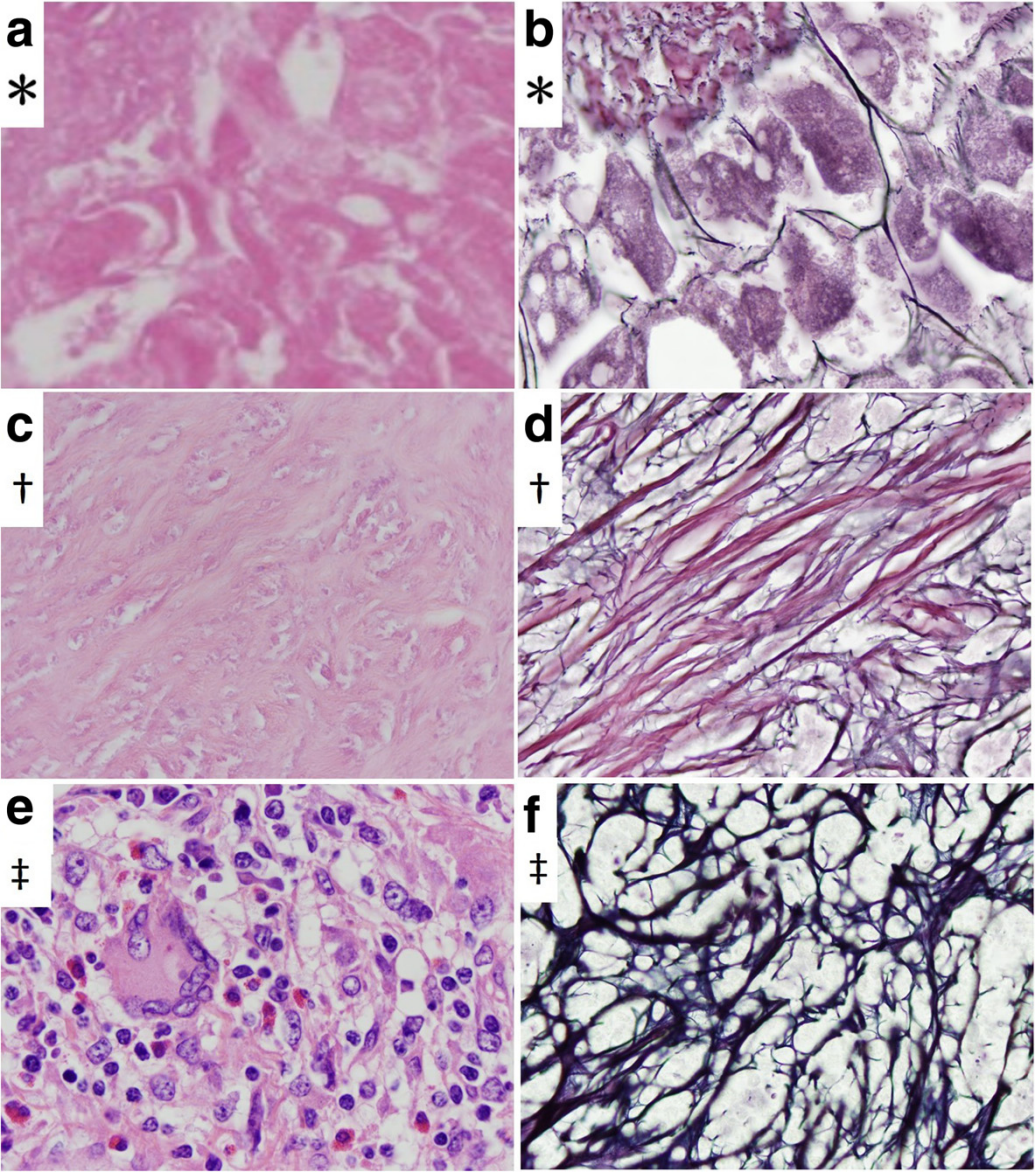
Histopathologic features. **a** Necrotic tissue (*, HE x 1000). **b** Silver staining revealed the necrotic tissue preserved the structure of the hepatic cell plate and involved the portal tract, suggesting that it is a result of necrosis of the hepatocytes (*, silver staining x 1000). **c** Fibrous tissue (**†,** HE x 1000). **d** Fibrous tissue (**†,** silver staining x 1000). **e** The outermost layer was the band of granuloma comprising macrophages of epithelioid feature cells, lymphocytes and plasma cells with some infiltration of eosinophils forming a distinct boundary between normal hepatic tissue and the necrotic region. Multinucleated giant cells were present but not prominent (**‡,** HE x 1000). **f** The outermost layer (**‡,** silver staining x 1000)

Macroscopic examination revealed that the resected liver measuring 90 x 35 x 20 mm in size contained a grey-white solid nodule measuring 23 x 20 mm in maximum diameter. Microscopic examination confirmed that the nodule consisted of coagulation necrosis in the center (Fig. 2, Fig. 3a,b). Silver staining revealed the necrotic tissue preserved the structure of the hepatic cell plate and involved the portal tract, suggesting that it resulted from necrosis of the hepatocytes (Fig. 3b). The necrotic region was encompassed with circular formation of dense fibrous tissue (desmoplastic area) (Fig. 3c,d).

**Fig. 4:**
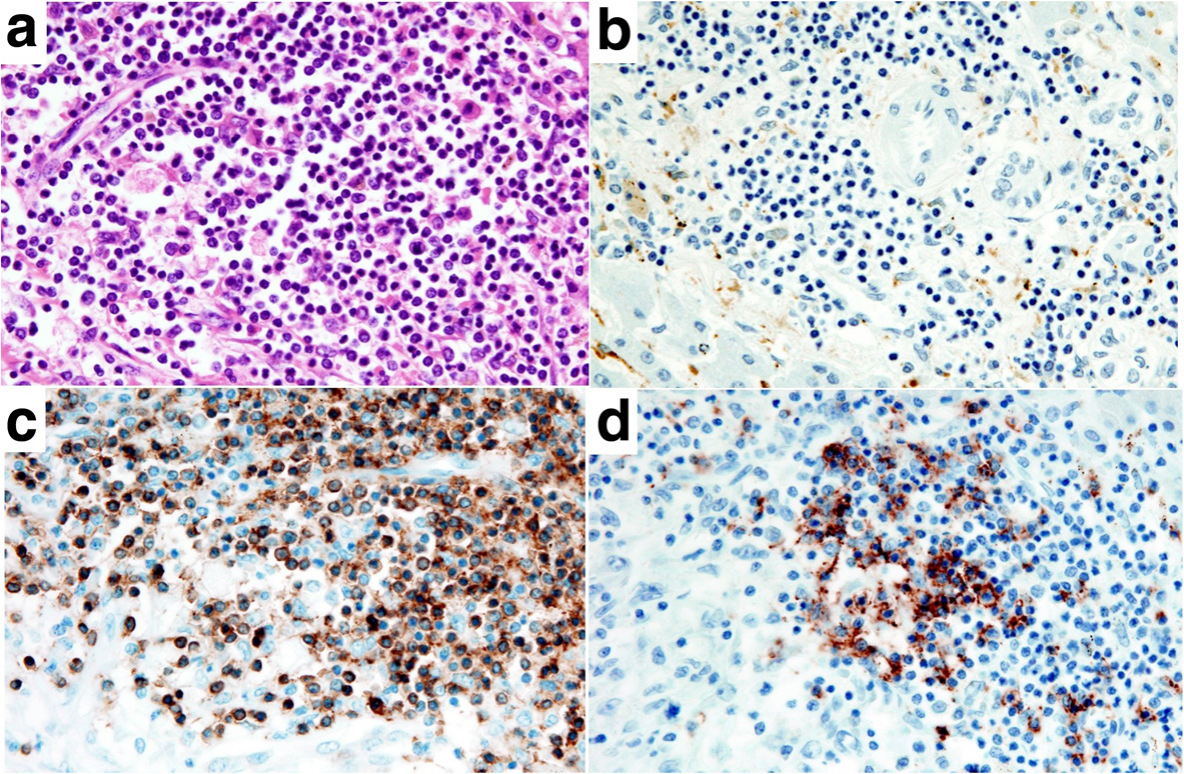
Immunohistochemistry (x 400). **a** HE. **b** CD68. **c** CD 3. **d** CD 20

The outermost layer was the band of granuloma comprising macrophages of epithelioid feature cells, lymphocytes and plasma cells with some of infiltration of eosinophils, which formed a distinct boundary between normal hepatic tissue and the necrotic region (Fig. 3e,f). Multinucleated giant cells were present but not prominent (Fig. 3e). There were no cancer cells, foamy leprae cells, eggs of a parasitic basis, Schaumann bodies, or asteroid bodies in the lesion. At the band of granuloma, immunohistochemistry revealed that there were many CD 3 positive cells and small amounts of CD 20 positive cells (Fig. 4). The ratio of CD 3 positive cells to CD 20 positive cells (indicating the T-cell/B-cell ratio) was 4:1.

Nineteen months after the latest operation, a mass developed again in the resected edge of the liver (the medial segment of the liver). A contrast-enhanced CT of the abdomen detected a 45-mm low-density mass without contrast enhancement involving a linear, high-density material. An abdominal MRI scan indicated the mass had a low signal intensity on T1-weighted images and a slight high intensity on T2-weighted images. There was a strand-like component lacking signal intensity in the middle of the mass. MR diffusion-weighted imaging depicted the mass with a high signal intensity. The patient was not given a gadolinium-contrast agent because of his renal failure. Sonazoid®-enhanced ultrasonography revealed an avascular and hypoechoic mass at Segment 4 in the plain phase, and no contrast enhancement was observed at any phase. The FDG-PET/CT scan was not performed. Finally, at twenty-three months after the latest operation, open medial segmentectomy of the liver and caudate lobectomy was conducted under suspicion of cancer recurrence because of an increase of the mass in the short term. Operators did not use additional metal staples or clips in this procedure.

**Fig. 5:**
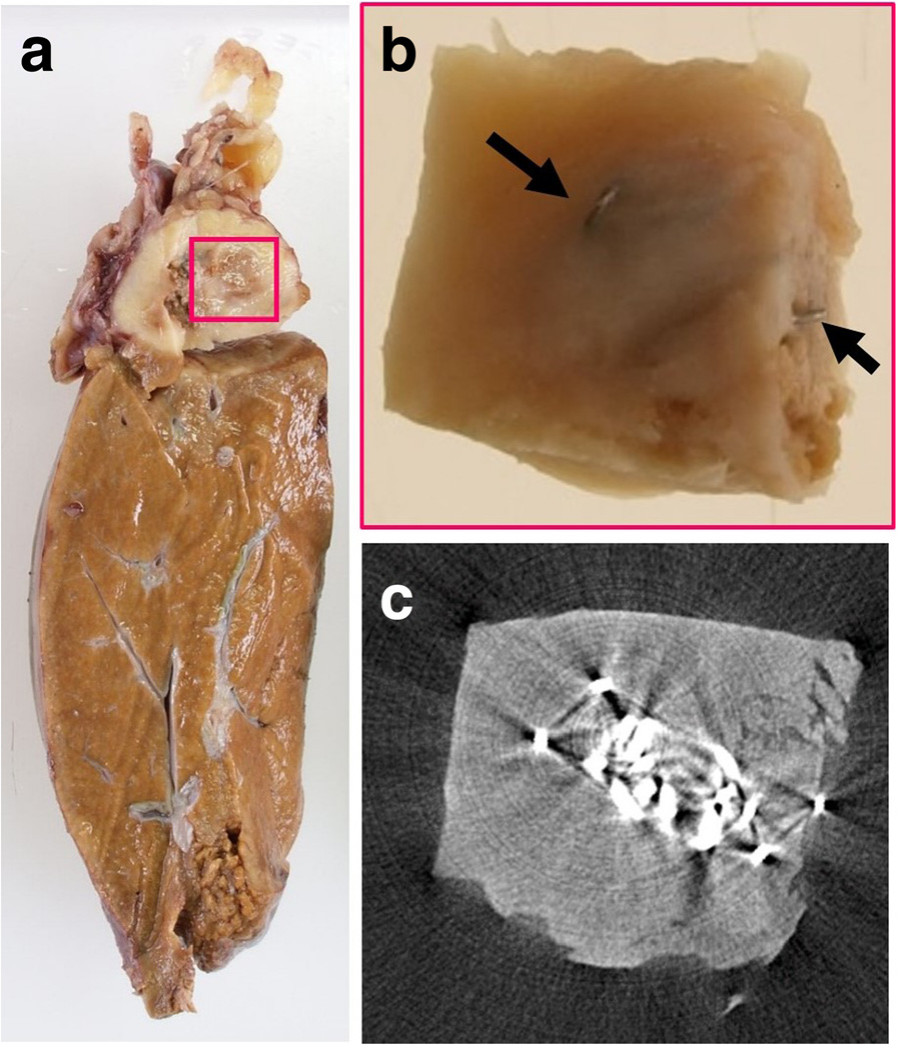
Macroscopic features. **a** A grey-white solid nodule measuring 45 x 38 mm in the resected liver. **b** Staple needles in the central portion of the nodule (arrows). **c** Micro CT

Gross examination revealed a grey-white solid nodule measuring 45 x 38 mm in maximum diameter containing staples in the middle of the resected specimen (Fig. 5a, b). A two-dimensional image gained by X-ray micro computed tomography (micro CT) depicted staple needles in the central portion of the nodule (Fig. 5c). Histopathological features of the staple-containing nodule are suggestive of granuloma as in the previous sample. Eosinophils were not prominent. As for the multinucleated giant cell, it appeared unclear. Both Ziehl-Nielsen staining and Grocott’s staining were negative.

Granuloma possibly due to retained staples was suspected on the basis of the histopathological findings and clinical course of the patient. To confirm sensitivity to titanium or other metals, the patient was subjected to a patch test. Titanium, vanadium and aluminum were all negative. The patient did not show any obvious recurrence in the liver 16 months postoperatively.

## Discussion

A search of the databases PubMed and Ichushi (in Japanese) using the query words “staple/stapler AND granuloma”, “clip/clipping AND granuloma”, “allergic granuloma” or “hepatic granuloma” yielded scattered reports involving the lung and granulomas possibly caused by surgical staples [2,3], but there were no articles referencing operations conducted for the liver.

Clips and staples applied for resection of the liver in our case were made of a titanium alloy (>90 % titanium, 2.0–3.0 % vanadium, 2.5–3.5 % aluminum). Titanium has been considered to be inert within the body [1], but several case reports have suggested that titanium may be a sensitizer when involving a pacemaker [4], orthopedic implants [5,6] or dental implants [7].

Although the method generally used to examine metal hypersensitivity is the epicutaneous test (so-called “patch test”), the patch test has been an examination procedure and mainly interprets the dermally-sensitized reaction and may not clearly reflect immune responses provoked by metal implants in deep tissue [12–16]. Moreover, since the test may itself induce sensitization as an irritant reaction, it can lead to a false positive or false negative result [6,13]. Furthermore, reproducibility varies due to several factors (e.g., site-to-site, inter-observer, etc.) [13,15].

Alternative testing methods have been utilized and include *in vitro* examinations such as the lymphocyte transformation test (LTT) or its modified version, the memory lymphocyte immunostimulation assay (MELISA®) [6,13,15]. However, there is debate among clinicians whether these tests are adequate diagnostic tools. Thus, there is currently no ideal test which is a reliable testing method for metal hypersensitivity [6,13]. Our diagnosis was therefore made comprehensively using patient history, clinical findings, and the results of the aforementioned tests in accordance with procedures used by other researchers [5].

Although there are several case reports of suspected hypersensitivity to titanium, the possibility remains of an allergic reaction induced by a small percentage of vanadium or aluminum released by corrosion of the titanium alloy [1]. In the present case, staples as a causative agent had already been removed by surgical intervention, and patch tests were all negative for titanium, vanadium and aluminum. We did not employ MELISA® testing because it was not readily available in Japan. Allergic response against metal has been known as one of the forms of delayed-type hypersensitivity (type IV allergy) associated with antigen-specific T cells, but we rarely encounter a granuloma that resulted from hypersensitivity to staples, especially those developed in the liver. Therefore, no reports have described the histopathological features of this condition in the liver [16]. However, it could easily be assumed that the infiltration pattern of lymphocytes in the lesion comprised dominant T cells, and this was verified by immunohistochemistry in this case. In addition, the fact that the band of granuloma included fewer multinucleated giant cells may suggest little concern with foreign body reaction for development of granuloma in this case.

## Conclusion

We report the first case of hepatic granuloma resulting from possible hypersensitivity to titanium alloy staples and/or clips. The three-layered structure consisting of necrosis containing titanium alloy, a desmoplastic (fibrous) area, and a band of granuloma could be interpreted as a histological characteristic for hepatic granuloma due to titanium alloy hypersensitivity.

## Consent

Written informed consent was obtained from the patient for publication of this Case Report and any accompanying images. A copy of the written consent is available for review by the Editor-in-Chief of this journal.

## References

[1] Steinemann SG (1998). Titanium-the material of choice?. Periodontol 2000.

[2] Tomita M, Matsuzaki Y, Edagawa M, Shimizu T, Masaaki H, Onitsuka T (2003). Pulmonary granuloma possibly caused by staples after video-associated thoracoscopic surgery. Ann Thorac Cardiovasc Surg.

[3] Yüksel M, Akgül AG, Evman S, Batirel HF (2007). Suture and stapler granulomas: a word of caution. Eur J Card-Thor Surg.

[4] Yamauchi R, Morita A, Tsuji T (2000). Pacemaker dermatitis from titanium. Contact Dermatitis.

[5] Thomas P, Bandl W-D, Maier S, Summer B, Przybilla B (2006). Hypersensitivity to titanium osteosynthesis with impaired fracture healing, eczema, and T-cell hyperresponsiveness *in vitro*: case report and review of the literature. Contact Dermatitis.

[6] Hallab N, Merritt K, Jacobs J (2001). Metal sensitivity in patients with orthopaedic implants. J Bone Joint Surg Am.

[7] Sicilia A, Cuesta S, Coma G, Arregui I, Guisasola C, Ruiz E (2008). Titanium allergy in dental implant patients: a clinical study on 1500 consecutive patients. Clin Oral Impl Res.

[8] Bircher AJ, Stern WB (2001). Allergic contact dermatitis from “titanium” spectacle frames. Contact Dermatitis.

[9] High WA, Ayers RA, Adams JR, Chang A, Fitzpatrick JE (2006). Granulomatous reaction to titanium alloy: An unusual reaction to ear piercing. J Am Acad Dermatol.

[10] Tamai K, Mitsumori M, Fujishiro S, Kokubo M, Ooya N, Nagata Y (2001). A case of allergic reaction to surgical metal clips inserted for postoperative boost irradiation in a patient undergoing breast conserving therapy. Breast Cancer.

[11] Hadedeya DS, Latif KA. A foreign body granuloma mimicking a tumor recurrence complicating a stapler site after a sigmoid cancer resection. World J Colorectal Surg. 2013;3. Iss. 1, Art 17.

[12] Merritt K, Brown SA (1996). Distribution of cobalt chromium wear and corrosion products and biologic reaction. Clin Orthop Relat Res.

[13] Valentine-Thon E, Müller K, Guzzi G, Kreisel S, Ohnsorge P, Sandkamp M (2006). LTT-MELISA® is clinically relevant for detecting and monitoring metal sensitivity. Neuro Endocrinol Lett.

[14] Müller K, Valentine-Thon E (2006). Hypersensitivity to titanium: clinical and laboratory evidence. Neuro Endocrinol Lett.

[15] Spiewak R (2008). Patch testing for contact allergy and allergic contact dermatitis. Open Allergy J.

[16] Stejskal V, Hudecek R, Stejskal J, Sterzl I (2006). Diagnosis and treatment of metal-induced side-effects. Neuro Endocrinol let.

